# Crohn’s and bones: assessing bone microarchitecture using high-resolution peripheral quantitative computed tomography

**DOI:** 10.1093/jbmrpl/ziaf190

**Published:** 2025-12-11

**Authors:** Rachel E Klassen, Cathy Lu, Steven K Boyd, Lauren A Burt

**Affiliations:** McCaig Institute for Bone and Joint Health, Cumming School of Medicine, University of Calgary, Calgary, AB T2N 4Z6, Canada; Department of Gastroenterology, Cumming School of Medicine, University of Calgary, Calgary, AB T2N 4Z6, Canada; McCaig Institute for Bone and Joint Health, Cumming School of Medicine, University of Calgary, Calgary, AB T2N 4Z6, Canada; McCaig Institute for Bone and Joint Health, Cumming School of Medicine, University of Calgary, Calgary, AB T2N 4Z6, Canada

**Keywords:** inflammatory bowel disease, BMD, DXA, bone quality, HR-pQCT

## Abstract

Crohn’s disease (CD) is known to negatively affect BMD and inflammatory (non-stricture) and stricturing CD are the two common phenotypes. Studies exploring CD phenotypes with advanced imaging techniques, such as HR-pQCT, are lacking. Therefore, the aim of this cross-sectional study was to examine differences in bone quality, including volumetric BMD, bone microarchitecture, bone geometry, estimated bone strength, and void spaces, between inflammatory and stricturing CD phenotypes using HR-pQCT. Participants aged 55 yr and over were recruited and scanned by HR-pQCT (XtremeCT II) at the distal radius and tibia. Finite element analysis estimated bone strength and void space analysis captured structural inhomogeneities in the trabecular compartment. Health history and CD-specific information were assessed with blood work and questionaries. Two-way ANOVA, with sex as a factor, compared CD phenotypes. Sixty-one participants were recruited (52% female, mean age: 65.3 ± 6.1 yr). Group-by-sex interactions were observed at the tibia. Specifically, males in the stricturing group exhibited a smaller cortical bone area than males in the inflammation group, while no differences were detected between groups for females (*p* = .038). Similarly, males in the stricturing group had lower cortical thickness than males in the inflammatory group, with no differences between groups for females (*p* = 0.033). At both the radius and tibia, there were group interactions for bone microarchitecture parameters where the stricturing group had compromised bone microarchitecture compared with the inflammatory group. No differences were observed for bone strength or fracture rate between groups. Using HR-pQCT, we observed differences in bone microarchitecture and geometry between individuals with inflammatory and stricturing CD phenotypes. Future research should be prospective and should consider CD phenotype and sex when assessing skeletal health and fracture risk in this population.

## Introduction

Crohn’s disease (CD) is an autoimmune disease characterized by chronic and acute inflammation of the gastrointestinal track.^1^ Canada has one of the highest global prevalences of CD, with incidence rates continuing to rise. As of 2023, approximately 410 per 100 000 Canadians were living with CD,^2^ and these rates appear to be lower in First Nations than the general population.^3^ In general, CD prevalence is higher in North American and European cohorts than Asian,^4^ and contribute to a significant burden on the healthcare system and affected individuals.^1^

**Figure 1:**
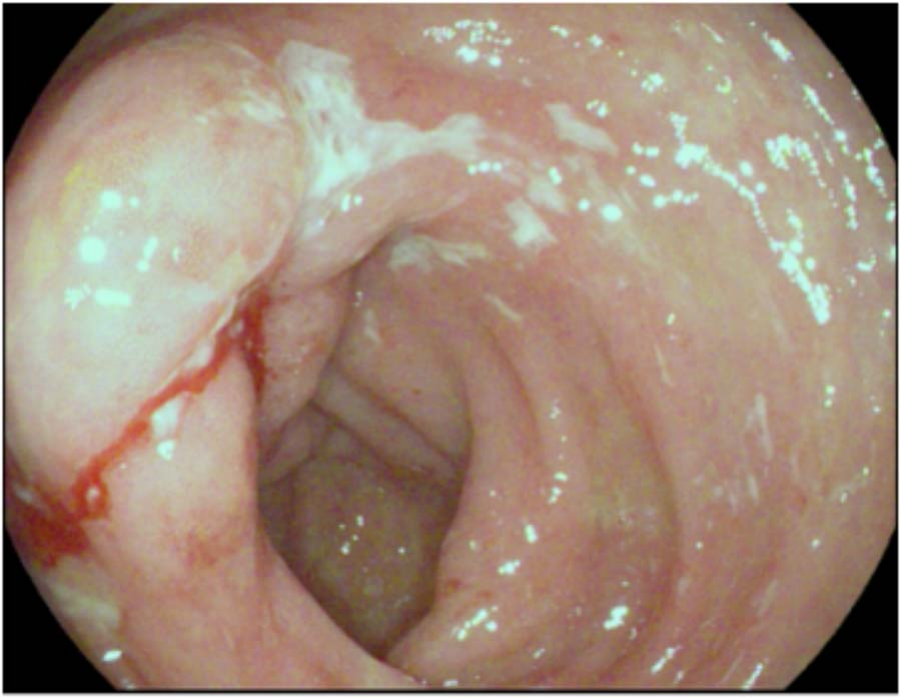
Strictured and ulcerated ileocecal valve in Crohn’s disease preventing entry into terminal ileum on colonoscopy.

Crohn’s disease has different presentations or phenotypes: stricturing disease due to fibrosis; penetrating disease due to fistulas between the gut and other structures; and disease lacking these features known as inflammatory or non-stricturing, non-penetrating disease; and stricturing, penetrating disease.^5^ Inflammatory CD is characterized by chronic, relapsing transmural inflammation of the gastrointestinal tract within the most distal portion of the small bowel, the terminal ileum, most regularly affected.^5^ Crohn’s disease phenotype can progress from inflammatory to stricturing, penetrating disease as repeated cycles of inflammation can lead to bowel damage.^5^ As bowel inflammation and damage occurs, narrowing of the bowel may lead to blockages (Figure 1), further negatively impacting quality of life.^5^

The age of CD diagnosis and phenotype have important implications for treatment decisions and long-term health outcomes. Pediatric-onset CD is often more aggressive and associated with impaired growth and reduced peak bone mass.^6–8^ In contrast, diagnosis in adulthood, particularly after the age of 40 yr, is more likely to be complicated by comorbidities and age-related bone loss. Across the lifespan, skeletal health is a critical concern in CD, influenced by factors, such as malabsorption, chronic inflammation, corticosteroid use, reduced physical activity, dietary restrictions, and bowel resections.^5^ For individuals with stricturing CD, self-imposed or medically recommended dietary limitations may exacerbate nutritional deficits, contributing to lower BMD. These disease-specific factors, combined with the effects of normal aging, place individuals with CD at increased risk of osteoporosis and fragility fractures.^9^ Furthermore, fragility fractures are associated with increased morbidity, functional decline, and mortality.^10^

HR-pQCT is a non-invasive imaging technology that assesses bone microarchitecture of the distal radius and tibia (nominal isotropic resolution of 61 μm), allowing for the quantification of volumetric BMD, bone geometry, microarchitecture, and strength estimates in vivo akin to a non-invasive bone biopsy.^11^ DXA is the clinical gold-standard for diagnostic bone health assessment and has been used to demonstrate lower BMD in individuals with CD,^12^ it is limited by its two-dimensional basis for measuring areal BMD (aBMD). Furthermore, HR-pQCT has an advantage over DXA, because it distinguishes between the cortical and trabecular compartments of bone and provides detailed microarchitectural information.^13^

Currently, there are 2 problems preventing the optimization of skeletal health in individuals with CD: (1) inadequate knowledge of the mechanisms underpinning skeletal fragility associated with CD and (2) the failure to identify individuals with CD who are at risk for low BMD. The aim of this study was to examine differences in bone quality, including volumetric BMD, bone microarchitecture, bone geometry, estimated bone strength, and void spaces between inflammatory and stricturing CD phenotypes using HR-pQCT. We hypothesized that individuals with stricturing CD would have lower volumetric BMD, compromised microarchitecture and geometry parameters, lower estimated bone strength, and larger, more numerous void spaces when compared against individuals with inflammatory CD, placing them at a higher risk for fracture.

## Materials and methods

### Participants

Patients were recruited from the University of Calgary Gastroenterology Clinic at Foothills Medical Center between April 2022 and September 2023. Inclusion criteria consisted of male and female patients with histologically confirmed CD, age greater than 55 yr (post-menopausal for females). Exclusion criteria included hormone replacement therapy, celiac disease, any gastrointestinal condition other than CD, cirrhosis, primary hyperparathyroidism, hypogonadism, short gut syndrome, ostomy, current or past eating disorder, current or past hyperthyroidism, esophageal or oropharyngeal dysphasia, premature menopause, CKD (stage 4/5/5D), previous osteoporosis treatment, including bisphosphonates, denosumab, and teriparatide, or prolonged mobility greater than 6 mo. Patients exhibiting fistulizing CD were excluded from our study. All patients included in this study were part of a larger study that excluded patients exhibiting enteric fistulizing behavior. This study was approved by the Conjoint Health Research Ethics Board (CHREB) at the University of Calgary (REB22-0014). All participants signed written consent prior to study enrollment.

### Anthropometric measurements and health history questionnaires

Height and weight were measured to the nearest 0.1 cm and 0.1 kg and used to calculate BMI (kg/m^2^). Using a health history questionnaire, participants self-reported bone-related events including fracture as well as current and previous medication usage and smoking history. Bowel resection status was captured from health history questionaries and confirmed with medical chart review.

### Crohn’s disease phenotype

Crohn’s disease activity, phenotype, and location were confirmed by gastroenterologists via diagnostic imaging (intestinal ultrasound and/or computed tomography enterography and/or magnetic resonance enterography) and pathological analysis from biopsies collected during routine standard of care colonoscopies. Patients with stricturing disease had increased bowel wall thickness >3 mm, luminal apposition <1 cm, and either fixed angulation suggesting chronic fibrosis, and/or prestenotic dilation. Patients with inflammatory disease may have had increased bowel wall thickness and luminal apposition; however, pre-stenotic dilation or a fixed angulation was not permitted. The Montreal Classification was employed to categorize age at diagnosis (A1, A2, and A3), location (L1, L2, L3, and L4), and behavior (B1, B2, and B3) to further characterize CD phenotype.^14^ Individuals with internal fistulizing or penetrating phenotype were excluded.^15^ Disease activity was assessed with the Harvey–Bradshaw index (HBI).^16^

### HR-pQCT imaging and analysis

Each participant underwent HR-pQCT (XtremeCT II, Scanco Medical AG) scans of their non-dominant distal radius and left tibia. If a previous fracture, surgery, or implant had been reported in the scan region, the opposite limb was scanned. The standard offset method was used where reference lines were placed at the distal endplates and scan acquisition began at 9.5 and 22.5 mm proximal to the endplate at the radius and tibia, respectively. A 10.2 mm of length from the endplate was scanned (168 slices) at an isotropic nominal resolution of 61 μm. Scans were individually evaluated for motion, scored from 1 to 5, with scans scoring 4 or 5 being removed from analyses.^17^

The manufacture’s standard evaluation protocol was used for image analysis. By a dual threshold technique, periosteal and endocortical contours were automatically generated and were manually inspected and corrected.^18^^,^^19^ Using greyscale images, volumetric BMD of the total, cortical, and trabecular regions (Tt.BMD, Ct.BMD, and Tb.BMD) were determined. Morphological microarchitecture variables included trabecular thickness (Tb.Th), trabecular separation (Tb.Sp), trabecular number (Tb.N), cortical thickness (Ct.Th), and cortical porosity (Ct.Po). All microarchitecture measurements were determined using direct measurement techniques from the segmented bone structure. Additionally, cross sectional total, trabecular, and cortical areas (Tt.Ar, Tb.Ar, and Ct.Ar), were determined. Our scanner was calibrated daily prior to use. Reproducibility in our lab ranges from 0.4% to 2.4% for volumetric BMD, 0.4%-1.2% for area, 0.8%-2.7% for trabecular microarchitecture variables, and 1.3%-2.9% and 1.6%-13.7% for Ct.Th and Ct.Po, respectively.^20^

To estimate bone strength, linear micro finite element analysis was calculated using the segmented HR-pQCT images with a voxel-by-voxel approach.^21^^,^^22^ Bone tissue properties of a Poisson’s ratio of 0.3 and a homogeneous Young’s modulus of 8748 MAp were assigned.^23^ An axial compression test with 1% compressive strain was used for model boundary conditions. Failure load (N) was estimated with the yield criterion of 2% critical volume and a critical strain of 0.7%.^24^ Models were solved using FAIM v8.0, (Numerics88 Solutions Ltd.).

Void space is a new, intuitive morphological parameter that captures localized bone loss in the trabecular microarchitecture.^25^ We applied this technique as a means of exploring the inhomogeneities of bone microarchitecture in individuals with CD. Specifically, void spaces were quantified in terms of number of void spaces (Vs.N), void space volume (Vs.V), and void space volume to total bone volume ratio (Vs.Tv).^25^ Large void spaces were defined as greater than 10% relative void space volume. The void space analysis script is available on GitHub https://github.com/PaediatricMSKImaging/Open_IPL/tree/main/voidspace.

### DXA imaging and analysis

All participants underwent DXA scans of the LS (L1-L4), left hip (femoral neck and total hip), and total body (Encore v16, GE Lunar, iDXA GE Healthcare). Scans were performed by one qualified technologist. Areal BMD (aBMD, g/cm^2^) and T-scores were obtained. In cases, where individual lumbar vertebrae, were excluded due to 1 or 2 vertebrae exhibiting artifacts, the aBMD was extrapolated using the reportable vertebrae. Localized LS results were excluded, if a study clinician deemed the area unreliable due to degeneration. FRAX risk estimating the 10-yr probability of a hip fracture and a major osteoporotic fracture (spine, forearm, hip, or shoulder fracture) were calculated.^26^

### Clinical laboratory tests

Table S1 outlines the clinical serum blood markers and fecal analyses (fecal calprotectin) collected as part of this study.

### Statistical analysis

Based on previous results for total vBMD using first-generation HR-pQCT, and exploring differences between CD and ulcerative colitis,^27^our a priori sample size was 52 participants (*n* = 26 per group), assuming 95% power, an error of 0.05, and an effect size of 0.939. As data was normally distributed, results are presented as means and SD for continuous variables or number and percentage for categorical variables. Tt.BMD and failure load results were compared with age- and sex-matched controls using freely available normative data (Normative, https://www.normative.ca) developed and maintained by the Bone Imaging Laboratory at the University of Calgary.^28^ T-tests and chi square compared differences between groups for descriptive and DXA data. Differences in bone quality between inflammatory and stricturing CD phenotypes were explored using a two-way ANOVA, with sex as a factor. Using the same statistical approach, 2 additional exploratory analyses were performed. We compared individuals with and without previous bowel resections, and age of CD diagnosis using the Montreal classification (<25 yr, 25-40 yr, and >40 yr), with a Tukey’s post hoc analysis. Statistical significance was defined as *p* < .05. Statistical analyses were run using SPSS (version 28).

## Results

A total of 61 participants were recruited and represent either inflammatory (*n* = 29) or stricturing (*n* = 32) CD phenotypes. Demographic and DXA data are presented in Table 1. The majority of our cohort was White (98%) and 52% were female. Age of diagnosis ranged from 13 to 73 yr for the inflammatory and 14 to 70 yr for the stricturing group. The number of participants with a history of previous bowel resection was higher for the stricturing (50%) compared with the inflammatory group (7%, *p* < .001). There were no other between group differences for demographic or DXA data. The mean HBI score was 2.5 for the inflammatory group (range: 0-9) and 2.6 for the stricturing group (range: 0-10). The number of participants with HBI score of ≤4 was 75% (*n* = 22) and 78% (*n* = 25) for the inflammatory and stricturing groups, respectively. About 41% (*n* = 25) of people had sustained a previous lifetime fracture, and 10 individuals sustained more than one fracture. Fracture rates did not differ significantly between groups. The most common reason for fracture was a slip, trip, or fall outside the home or a sporting-related fracture. One fracture was the result of a fall from sitting height or lower. By DXA classification, 44% of the cohort had low bone mass (osteopenic) and 16% were osteoporotic. Specifically, 10% (*n* = 3) of the inflammatory and 19% (*n* = 6) of the stricturing group had osteoporosis.

**Table 1:** Descriptive and DXA variables of the Crohn’s disease (CD) cohort by phenotype.

**Variable**	**Inflammatory CD** **(*n* = 29)**	**Stricturing CD** **(*n* = 32)**
	**Mean (SD)**	**Mean (SD)**
**Age (yr)**	64.65 ± 6.64	65.84 ± 5.67
**Age of diagnosis (yr)**	47.52 ± 14.48	41.00 ± 17.44
**Height (cm)**	169.45 ± 7.65	169.48 ± 9.29
**Weight (kg)**	82.43 ± 14.65	78.72 ± 17.58
**Body mass index (kg/m^2^)**	28.71 ± 4.95	27.41 ± 6.07
**Sex**	***N*** **(%)**	***N*** **(%)**
** Male**	15 (51.7)	14 (43.8)
** Female**	14 (48.3)	18 (56.3)
**Previous bowel resection(s)**	2 (6.9)	16 (50)
**Former smoker**	13 (44.8)	14 (43.8)
**Current smoker**	2 (6.9)	5 (15.6)
**Previous fracture** ^**a**^	10 (40.0)	15 (55.5)
**Previous steroid use**	5 (17.2)	8 (25.0)
**Current treatment**		
** None**	8 (27.6)	7 (21.8)
** Steroids**	1 (3.4)	1 (3.1)
** DMARDS**	7 (24.1)	1 (3.1)
** Biologic**	13 (44.8)	23 (71.9)
**DXA variables**	**Mean (SD)**	**Mean (SD)**
**Visceral adipose tissue area (cm^2^)**	227.51 ± 115.65	178.68 ± 125.42
**Lumbar spine aBMD (g/cm^2^)** ^**b**^	1.133 ± 0.177	1.056 ± 125.42
**Lumbar spine T-score** ^**b**^	−0.372 ± 1.499	−1.006 ± 0.179
**Femoral neck aBMD (g/cm^2^)** ^**c**^	0.917 ± 0.113	0.888 ± 0.140
**Femoral neck T-score** ^**c**^	−0.871 ± 0.816	−1.081 ± 1.005
**Total hip aBMD (g/cm^2^)** ^**c**^	0.974 ± 0.132	0.925 ± 0.167
**Total hip T-score** ^**c**^	−0.254 ± 1.045	−0.656 ± 1.328
**FRAX major osteoporotic fracture** ^**d**^	6.836 ± 3.693	9.113 ± 1.328
**FRAX hip fracture** ^**d**^	1.025 ± 1.392	1.613 ± 5.499

Results are presented as number of participants with percentage in parentheses for categorical data or mean and SD for continuous data as indicated.

Previous fracture: represents any lifetime fracture occurrence. Abbreviations: aBMD, areal BMD; FRAX, fracture risk assessment tool.

a Fracture data was missing for 4 inflammatory and 5 stricturing CD participants.

^b^ One participant from the inflammatory group had bilateral hip replacements and therefore does not have hip DXA data.

c One participant from the stricturing group had LS degeneration and therefore does not have spine DXA data.

d One participant from each group was removed from FRAX.

Regarding the laboratory values, albumin was significantly different between groups (*p* = .017), where the stricturing group (36.78 ± 3.50 g/L) had lower serum albumin than the inflammatory group (38.72 ± 2.25 g/L). No other biomarkers were significantly different between groups (Table S1). Two males (one in each group) had testosterone values <8 nmol/L meeting the typically used diagnostic threshold for hypogonadism.

One radius and one tibia HR-pQCT scan were removed due to motion artifact (motion score of 4, stricturing group for both). The HR-pQCT results for Tt.BMD and failure load, compared with age- and sex-matched normative data are shown in the supplementary material (Figures S1-S8). At the radius, 6 females and 6 males had Tt.BMD below the 10th percentile, compared with normative data. Similarly, at the radius, 4 females and 5 males had estimated failure load below the 10th percentile, compared with normative data. Comparable results for Tt.BMD and failure load were observed at the tibia. While individuals with CD had below average Tt.BMD and failure load, the spread of the data was large and not all individuals with CD have low Tt.BMD and failure load.

HR-pQCT results comparing the CD phenotypes are summarized in Table 2. At the radius, there were no group-by-sex interactions. However, there were group interactions for bone microarchitecture, geometry, and void space number, where the stricturing group had lower Ct.Th, larger Tt.Ar, and Tb.Ar with a higher number of void spaces compared with the inflammatory group at the radius. As expected, there were sex interactions for all variables at the radius with the exception of Ct.BMD, Ct.Po, and all 3 void space variables.

**Table 2:** HR-pQCT results for the Crohn’s disease phenotypes at the distal radius and tibia.

	**Inflammatory** **(*n* = 29)**	**Stricturing** **(*n* = 31)**	***p*-value**
**Radius**			
**Total vBMD (mg HA/cm^3^)**	319.60 ± 69.36	279.1 ± 83.53	.054
**Failure load (N)**	3115.44 ± 945.37	2853.60 ± 1108.40	.316
**Cortical vBMD (mg HA/cm^3^)**	890.98 ± 62.52	862.03 ± 86.46	.177
**Trabecular vBMD (mg HA/cm^3^)**	159.90 ± 36.35	145.09 ± 46.09	.216
**Trabecular thickness (mm)**	0.239 ± 0.020	0.239 ± 0.019	.845
**Trabecular separation (mm)**	0.744 ± 0.200	0.881 ± 0.368	.103
**Trabecular number (1/mm)**	1.34 ± 0.22	1.22 ± 0.30	.082
**Cortical thickness (mm)**	1.061 ± 0.249	0.925 ± 0.253	**.035**
**Cortical porosity (%)**	0.802 ± 0.561	0.796 ± 0.570	.845
**Total area (mm^2^)**	283.70 ± 66.84	307.97 ± 75.10	**.028**
**Cortical area (mm^2^)**	62.70 ± 17.68	56.32 ± 16.14	.116
**Trabecular area (mm^2^)**	224.80 ± 59.32	255.54 ± 72.79	**.015**
**Number of void spaces**	0.31 ± 0.54	0.78 ± 1.01	**.041**
**Void space volume (mm^3^)**	46.53 ± 146.53	108.75 ± 176.32	.187
**Void space volume to total bone volume (%)**	1.48 ± 4.45	3.53 ± 5.96	.188
**Tibia**			
**Total vBMD (mg HA/cm^3^)**	297.60 ± 61.89	267.50 ± 67.85	.087
**Failure load (N)**	8398.63 ± 2067.71	7880.55 ± 2374.30	.369
**Cortical vBMD (mg HA/cm^3^)**	842.20 ± 71.01	814.6 ± 88.91	.238
**Trabecular vBMD (mg HA/cm^3^)**	164.40 ± 32.82	157.00 ± 46.21	.595
**Trabecular thickness (mm)**	0.263 ± 0.026	0.264 ± 0.030	.858
**Trabecular separation (mm)**	0.783 ± 0.116	0.893 ± 0.321	.110
**Trabecular number (1/mm)**	1.262 ± 0.176	1.190 ± 0.280	.309
**Cortical thickness (mm)**	1.554 ± 0.345	1.378 ± 0.309	**.033**
**Cortical porosity (%)**	3.38 ± 1.89	3.49 ± 1.51	.947
**Total area (mm^2^)**	721.1 ± 146.90	772.10 ± 138.69	.060
**Cortical area (mm^2^)**	141.02 ± 33.42	127.19 ± 29.12	.050
**Trabecular area (mm^2^)**	585.5 ± 134.93	650.50 ± 139.97	**.033**
**Number of void spaces**	0.62 ± 0.78	0.74 ± 0.77	.572
**Void space volume (mm^3^)**	52.26 ± 99.90	232.96 ± 466.03	.053
**Void space volume to total bone volume (%)**	0.74 ± 1.42	2.92 ± 5.24	**.044**

Data are presented as mean and SDs. The *p*-value is the main effect for group from the two-way ANOVA. Bold text indicates significance between groups.

Abbreviation: vBMD, volumetric BMD.

**Figure 2:**
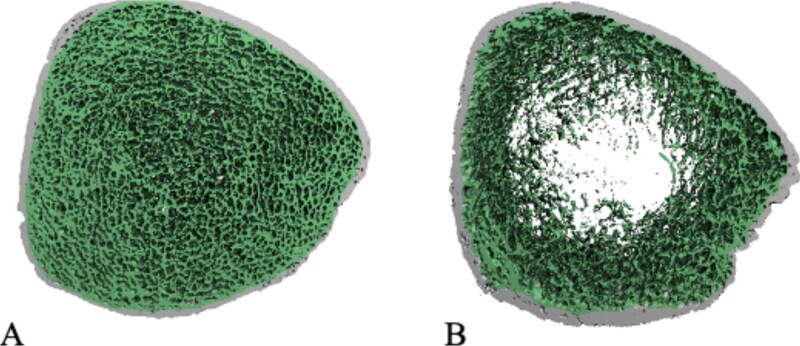
HR-pQCT tibial cross-sectional images representing individuals with Crohn’s disease: (A) without a void space and (B) with a large void space.

At the tibia, there were 2 group-by-sex interactions, where males in the stricturing group had smaller Ct.Ar than males in the inflammation group; however, there were no difference between groups for females (*p* = .038). Similarly, males in the stricturing group had lower Ct.Th than males in the inflammatory group, with no difference for females (*p* = .033). Group interactions at the tibia were observed for trabecular bone geometry and void space volume to total bone volume, where the stricturing group had bigger bones (Tb.Ar) with larger void space volume to total volume than the inflammatory group (Figure 2). Furthermore, sex interactions at the tibia were observed for Tt.BMD, Ct.BMD, Tb.BMD, failure load, Ct.Po, Tb.Th, and Tt.Ar.

At the radius, our exploratory analysis found that individuals with previous bowel resections had smaller Ct.Ar compared with those who were bowel resection naïve (*p* = .024, Table S2). Furthermore, at the tibia, there were group-by-sex-interactions for Tb.N and Tb.Sp, where females in the bowel resection group had lower Tb.N (*p* = .035) and higher Tb.Sp (*p* = .044) than females in the bowel resection naïve group, with no between group differences for males.

Our exploratory analysis for age of diagnosis revealed lower Ct.BMD (*p* = .005) and Ct.Th (*p* = .045) for individuals who were diagnosed with CD between 25 and 40 yr compared with those diagnosed before 25 yr at the radius. There were no between group differences at the tibia (Table S3).

## Discussion

This study examined the differences in skeletal health between individuals with inflammatory and stricturing CD phenotypes using HR-pQCT. Individuals with stricturing CD had lower cortical thickness with larger bone area, both total and trabecular, as well as a compromised trabecular region, shown through void space analysis, compared to individuals with inflammatory CD. No group differences in BMD, estimated bone strength or fracture incidence were observed. Based on these results, we hypothesize that pathological endosteal resorption is more likely occurring than homeostatic periosteal remodeling in individuals with CD, and that this effect is further enhanced in individuals with stricturing CD.

To our knowledge, this is the first study to examine the differences between CD phenotypes using HR-pQCT. Previous studies have examined inflammatory bowel disease (IBD), an umbrella term for CD and ulcerative colitis, using HR-pQCT.^27^^,^^29^^,^^30^ While similar, previous studies compared IBD cohorts with healthy controls^27^^,^^29^^,^^30^ and performed sub-analyses based on fracture history,^29^ or IBD classification (CD to ulcerative colitis).^27^^,^^30^ All HR-pQCT studies reported bone deficits for individuals with IBD compared with controls. These previous studies explored the bone health of individuals with mean age of 23^29^^,^^30^ or 44 yr,^27^ compared with our older adults aged 65 yr. Previously, individuals with IBD have been shown to have lower total,^27^^,^^29^^,^^30^ cortical,^27^ and trabecular^29^ volumetric BMD as well as detriments to the trabecular microarchitecture.^27^^,^^29^^,^^30^ Bone strength was not explored in any of the previous HR-pQCT studies. While we did not report between group differences in volumetric BMD in our older cohort, we observed trends of 10%-13% lower total volumetric BMD for individuals with stricturing compared with inflammatory CD. With a larger sample size, we assume group differences would have emerged, with lower total volumetric BMD in individuals with stricturing compared with inflammatory CD.

Our primary analysis did not observe group differences in standard HR-pQCT trabecular parameters (eg, Tb.N, Tb.Th, and Tb.Sp); however, we report group differences in trabecular bone through an advanced technique, known as void space analysis, designed to capture inhomogeneities within the trabecular bone compartment. Void spaces are indicative of important trabecular irregularities that cannot be measured using traditional morphometric parameters and can dramatically alter the structure and strength of the bones in CD patients (Figure 2). These localized bone deteriorations have been found to be systemic and increase with age.^25^ Currently, the development of void spaces are unknown and additional research should attempt to determine if they are pathological in nature.^25^ Void spaces are clinically relevant because they are unlikely to be reversed through typical bone remodeling mechanisms including anti resorption therapies and interventions.^25^ Previously, void spaces have not been well captured by standard HR-pQCT analyses, and this advanced method was not available at the time of other HR-pQCT studies.^27^^,^^29^ It is possible the larger void space volume to total bone volume we observed in the stricturing group was the result of previous bowel resection as there was a higher prevalence of bowel resection in the stricturing (50%) than the inflammatory (7%) group. Void space only emerge in the trabecular compartment and therefore could be the underlying reason that previous CD studies, report lower density and microarchitecture of trabecular bone.^27^^,^^29^^,^^30^

Like other IBD cohorts,^27^^,^^29^^,^^30^ we observed lower cortical thickness in those with stricturing than inflammatory CD. However, in addition to the differences between groups for cortical thickness, we report a group-by-sex effect at the tibia, where males in the stricturing group had lower cortical thickness than males in the inflammatory group, with no differences between groups for females. This differs from previous work reporting female sex to be associated with poorer bone quality in individuals with IBD^27^ and aligns with large retrospective study that found males are more likely to have low BMD in those with CD.^31^ Interestingly, males with spondyloarthritis, a disease that typically causes inflammation to the spine and is common in individuals with CD, display deterioration to cortical bone microarchitecture, compared with females who tend to have defects in the trabecular compartment.^32^ Through the combination of our primary and exploratory analysis, investigating differences between individuals with and without previous bowel resection, we support these sex differences to the trabecular and cortical bone compartments in individuals with CD. Males with stricturing CD were driving the differences we observed in cortical microarchitecture, whereas females with previous bowel resection had deficiencies to their trabecular microarchitecture. Collectively, these results suggest sex-specific bone adaptations may occur in individuals with CD and that previous bowel resection might influence bone microarchitecture; however, future longitudinal studies are required to confirm.

It is well known that individuals with CD have a higher prevalence of osteoporosis and fracture risk than the general population.^33^ While 44% of our cohort had low bone mass and 16% were osteoporotic, we did not observe group differences in areal BMD between CD phenotypes; although, the stricturing group did trending toward lower areal BMD than the inflammatory group. In contrast, HR-pQCT observed differences between stricturing and inflammatory CD for cortical bone microarchitecture and bone size. Imaging with HR-pQCT allows for compartment-specific analysis of bone, which has been shown to have greater sensitivity when measured against DXA at the same anatomical site.^34^ It is possible that differences are occurring between the CD phenotypes; however, they are below the observable threshold for DXA.

Crohn’s disease is characterized by strongly enhanced endocortical resorption, resulting in an enlargement of the medullary cavity, decreased cortical thickness, and loss of trabecular bone.^27^ Within the limitations of a cross-sectional study, we support this statement particularly for individuals with stricturing CD. Compared with other studies^29^^,^^30^ few participants in our study were diagnosed with CD in childhood. Our exploratory analysis investigating age of diagnosis is limited by a small sample size and should be interpreted with caution. The older age of diagnosis (over 40 yr) in our cohort compared with others could explain some of the differences observed between studies.^27^^,^^29^^,^^30^ Most participants (92%) in our study completed growth and development, including peak bone mass, without CD. This likely reduced the cumulative impact of chronic inflammation and corticosteroid exposure on the developing skeleton, which in early-onset disease has been associated with impaired linear growth, delayed puberty, reduced peak bone mass accrual, and increased fracture risk.^6^^,^^8^^,^^35^^,^^36^ Furthermore, delayed diagnosis of CD, which is not uncommon in adult-onset CD,^37^ may have led to underestimation of true disease duration and cumulative inflammatory burden, potentially biasing the observed associations with BMD.

We did not report between group differences in standard trabecular bone analyses between CD phenotypes, possibly due to similar disease states, nutritional status, relatively low use of steroids over the last 12 mo and low disease severity, as indicated by 77% of individuals having a HBI score ≤4. These factors are likely to influence the trabecular more than cortical bone as it is more metabolically active. Specifically, Haschka and colleagues report 50% of participants with a history of chronic high-dose glucocorticoid use and a median HBI score of four which may explain the trabecular compartment differences observed between CD and controls.^27^

The participants within our study were relatively healthy. They were vitamin D sufficient, PTH did not appear to be suppressed, allowing for adequate bone formation, and they had no history of previous osteoporosis treatment. While albumin was different between groups and lower in the stricturing group, possibly indicating lower nutrient absorption and inflammation in the digestive tract, the group mean was within normal values. However, the results of this study were likely impacted by various disease associated factors, such as differences in diet between the CD phenotypes and variances in absorption due to disease activity and/or bowel resections. This study did not examine lifestyle factors, such as the effect of physical activity, duration of therapeutic medications, and the number of previous CD flares, all of which have the ability to affect bone. Although, we collected data on current steroid use and use over the previous 12 mo, we were unable to capture earlier lifetime exposure. As corticosteroid-associated bone loss can be cumulative and occur with short-term use, prior unrecorded use may have influenced our results and introduced confounding founders in our analyses. Osteoimmunology crosstalk between the disease mediated inflammatory cytokines and bone metabolism could have influenced our findings^38^^,^^39^ and proinflammatory cytokines, mediated by CD activity, may be interfering with homeostatic metabolism of bone, creating an osteologic imbalance in remodeling and resorption.^40^ Finally, we did not measure limb length in our study and performed HR-pQCT imaging using the fixed offset method. Therefore, there may have been a small number of participants in our study with shorter limbs who were scanned at a different anatomical region; however, both the fixed and relative offset methods are influenced by the intrinsic variation of bone anatomy with respect to limb length and metaphyseal region.^41^

Future studies should use HR-pQCT to explore changes in skeletal health for individuals with CD, to determine the mechanisms underpinning skeletal fragility and rate of bone loss. Furthermore, comparisons should include age- and sex-matched individuals without CD. Biologic therapies and their impact on bone in this unique population should also be investigated. Over half the participants in our study were currently taking biologic medication. Unlike steroids, biologic therapies may offer a somewhat protective mechanism for bones as they suppress the inflammatory cascade involved in osteoclastic activity.^42^ For example, TNF-alpha (TNF-α) antagonists may provide osteologic protection as TNF-α has direct effects on the RANK/RANKL/OPG pathway of bone homeostasis.^43^

## Conclusion

We have shown differences in skeletal health between individuals with inflammatory and stricturing CD phenotypes using HR-pQCT, where the stricturing group had compromised bone microarchitecture compared with the inflammatory group. Crohn’s disease phenotype and sex should be considered when assessing skeletal health and fracture risk in this population. By identifying individuals with CD who have poor bone microarchitecture clinicians may be able to stratify patients for appropriate diet and bone interventions, preventing fragility fracture occurrence and maintaining quality of life in individuals with CD.

## Supplementary Material

Supplementary_materials_ziaf190

## Data Availability

The authors commit to making anonymized data that support the findings of this study available upon reasonable request. To gain access, data requestors will need to sign a data access agreement.
