# Supplementary material for: Crohn’s and bones: assessing bone microarchitecture using high-resolution peripheral quantitative computed tomography
Source: JBMR Plus. 2025 Dec 11;10(2):ziaf190. doi: 10.1093/jbmrpl/ziaf190 (PMC12790273; doi:10.1093/jbmrpl/ziaf190)
Supplement: Supplementary_materials_ziaf190 [file supplementary_materials_ziaf190.pdf]

Supplementary Table 1. Laboratory values for the Crohn's Disease cohort by phenotype.

	**Inflammatory (n=29)**	**Stricturing (n=32)**	**P-Value**
Fecal Calprotectin (mcg/g) [a]	278.52±455.32	138.64±105.89	0.259
C-Reactive Protein (mg/L)	3.10±2.56	2.85±4.09	0.784
Calcium (mmol/L)	2.39±0.1	2.37±0.09	0.581
25-Hydroxy Vitamin D (nmol/L)	100.5±36.83	95.41±47.12	0.742
Parathyroid Hormone (ng/L)	31.66±12.87	35.52±17.61	0.368
Albumin (g/L)	38.72±2.25	36.78±3.50	**0.017**
Total Bilirubin (umol/L)	10.72±5.28	11.75±16.18	0.755
Alkaline Phosphatase (U/L)	88.41±24.95	81.66±29.62	0.294
Alanine Aminotransferase (U/L)	32.31±32.44	25.19±12.74	0.244
Gamma Glutamyl Transferase (U/L)	32.41±37.62	21.83±16.45	0.149
Creatinine (umol/L)	82.45±25.53	81.75±28.39	0.880
Testosterone (nmol/L) [b]	15.02±5.30	17.68±8.62	0.354

Data are presented as mean and standard deviations. The p-value is the main effect for group from the two-way ANOVA. Bold text indicates significance between groups.

[a] Inflammatory group n=13; Stricturing group n=10.

[b] Male individuals only, n=13 per group.

Supplementary Table 2. High resolution peripheral quantitively computed tomography results for Crohn's Disease bowel resection status at the distal radius and tibia.

	Bowel Resection Naïve (n=42)	Previous Bowel Resection (n=18)	P-Value
**Radius**			
Total vBMD (mg HA/cm^3)	310.70±81.10	270.77±68.05	0.113
Failure Load (N)	3155.57±1028.14	2587.63±950.10	0.062
Cortical vBMD (mg HA/cm^3)	886.31±72.08	852.00±83.46	0.129
Trabecular vBMD (mg HA/cm^3)	157.81±41.27	139.24±41.87	0.246
Trabecular Thickness (mm)	0.240±0.019	0.236±0.021	0.903
Trabecular Separation (mm)	0.797±0.321	0.858±0.264	0.735
Trabecular Number (1/mm)	1.30±0.270	1.21±0.269	0.411
Cortical Thickness (mm)	1.034±0.268	0.889±0.204	0.061
Cortical Porosity (%)	0.804±0.563	0.786±0.569	0.755
Total Area (mm^2)	302.34±74.13	282.00±65.35	0.698
Cortical Area (mm^2)	62.57±17.37	52.01±14.14	**0.024**
Trabecular Area (mm^2)	243.65±70.75	233.70±61.92	0.915
Number of Void Spaces	0.49±0.83	0.72±0.89	0.591
Void Space Volume (mm^3)	80.00±172.58	77.06±148.12	0.772
Void Space Volume to Total Bone Volume (%)	2.48±5.52	2.74±5.09	0.920
**Tibia**			
Total vBMD (mg HA/cm^3)	287.98±72.16	266.98±46.72	0.319
Failure Load (N)	8350.38±2403.21	7603.56±1650.93	0.280
Cortical vBMD (mg HA/cm^3)	838.28±84.30	801.70±68.47	0.142
Trabecular vBMD (mg HA/cm^3)	165.65±41.97	147.79±32.75	0.177
Trabecular Thickness (mm)	0.262±0.027	0.267±0.030	0.529

Trabecular Separation (mm)	0.806±0.245	0.927±0.246	0.166
Trabecular Number (1/mm)	1.265±0.233	1.124±0.239	0.086
Cortical Thickness (mm)	1.462±0.370	1.467±0.240	0.845
Cortical Porosity (%)	3.18±1.59	4.09±1.79	0.117
Total Area (mm^2)	758.47±151.42	718.66±121.86	0.525
Cortical Area (mm^2)	134.88±34.97	131.33±22.49	0.926
Trabecular Area (mm^2)	629.48±148.95	592.68±115.21	0.554
Number of Void Spaces	0.63±0.82	0.82±0.63	0.435
Void Space Volume (mm^3)	128.04±390.23	190.09±229.37	0.694
Void Space Volume to Total Bone Volume (%)	1.51±4.22	2.78±3.39	0.416

Data are presented as mean and standard deviations. The p-value is the main effect for group from the two-way ANOVA. Bold text indicates significance between groups.

vBMD: volumetric bone mineral density

Supplementary Table 3. High resolution peripheral quantitively computed tomography results for Crohn's Disease age at diagnosis classification at the distal radius and tibia.

	A1<br><25 years old<br>(n=11)	A2<br>25-40 years old<br>(n=9)	A3<br>>40 years old<br>(n=40)
**Radius**			
Total vBMD (mg HA/cm^3)	312.19±87.76	327.76±59.59	308.74±75.72
Failure Load (N)	3218.52±1088.33	2186.63±619.38	3099.29±1024.76
Cortical vBMD (mg HA/cm^3)	913.71±61.52	819.15±76.41 [a]	878.45±74.55
Trabecular vBMD (mg HA/cm^3)	148.06±58.71	133.08±38.83	157.70±36.79
Trabecular Thickness (mm)	0.246±0.023	0.238±0.012	0.237±0.021
Trabecular Separation (mm)	0.870±0.306	1.049±0.539	0.748±0.196
Trabecular Number (1/mm)	1.209±0.324	1.103±0.364	1.338±0.213
Cortical Thickness (mm)	1.057±0.260	0.739±0.129 [a]	1.029±0.251
Cortical Porosity (%)	0.45±0.24	0.77±0.42	0.90±0.62
Total Area (mm^2)	294.98±36.44	273.44±60.28	301.72±80.84
Cortical Area (mm^2)	63.80±15.86	42.55±9.44	61.99±16.73
Trabecular Area (mm^2)	235.01±36.98	234.52±56.80	243.61±76.99
Number of Void Spaces	0.64±0.67	1.11±1.05	0.41±0.81
Void Space Volume (mm^3)	130.30±223.88	163.16±232.21	46.96±117.97
Void Space Volume to Total Bone Volume (%)	4.08±6.74	5.75±8.24	1.45±3.73
**Tibia**	**(n=11)**	**(n=8)**	**(n=41)**
Total vBMD (mg HA/cm^3)	291.68±68.68	226.89±38.16	290.21±65.87
Failure Load (N)	8515.38±2023.72	6306.39±1547.16	8396.41±2255.28
Cortical vBMD (mg HA/cm^3)	854.21±80.16	758.86±52.47	834.35±80.44
Trabecular vBMD (mg HA/cm^3)	164.16±51.19	139.40±26.98	163.77±38.61
Trabecular Thickness (mm)	0.269±0.034	0.263±0.037	0.263±0.025

Trabecular Separation (mm)	0.816±0.158	1.016±0.334	0.812±0.242
Trabecular Number (1/mm)	1.227±0.236	1.082±0.339	1.253±0.218
Cortical Thickness (mm)	1.507±0.225	1.194±0.239	1.5056±0.358
Cortical Porosity (%)	3.36±2.14	4.21±1.72	3.32±1.56
Total Area (mm^2)	742.72±92.28	734.66±115.26	751.21±161.25
Cortical Area (mm^2)	137.65±20.52	107.25±28.16	138.06±32.90
Trabecular Area (mm^2)	610.52±92.88	632.85±99.11	618.65±158.39
Number of Void Spaces	0.91±0.94	1.00±0.58	0.56±0.74
Void Space Volume (mm^3)	104.80±149.73	322.97±315.94	121.97±389.51
Void Space Volume to Total Bone Volume (%)	1.42±2.12	4.46±4.29	1.48±4.24

Data are presented as mean and standard deviations. Significant differences between groups represent main effect for group from the two-way ANOVA.

[a] Significantly ($p<0.05$) lower than A1.

vBMD: volumetric bone mineral density;

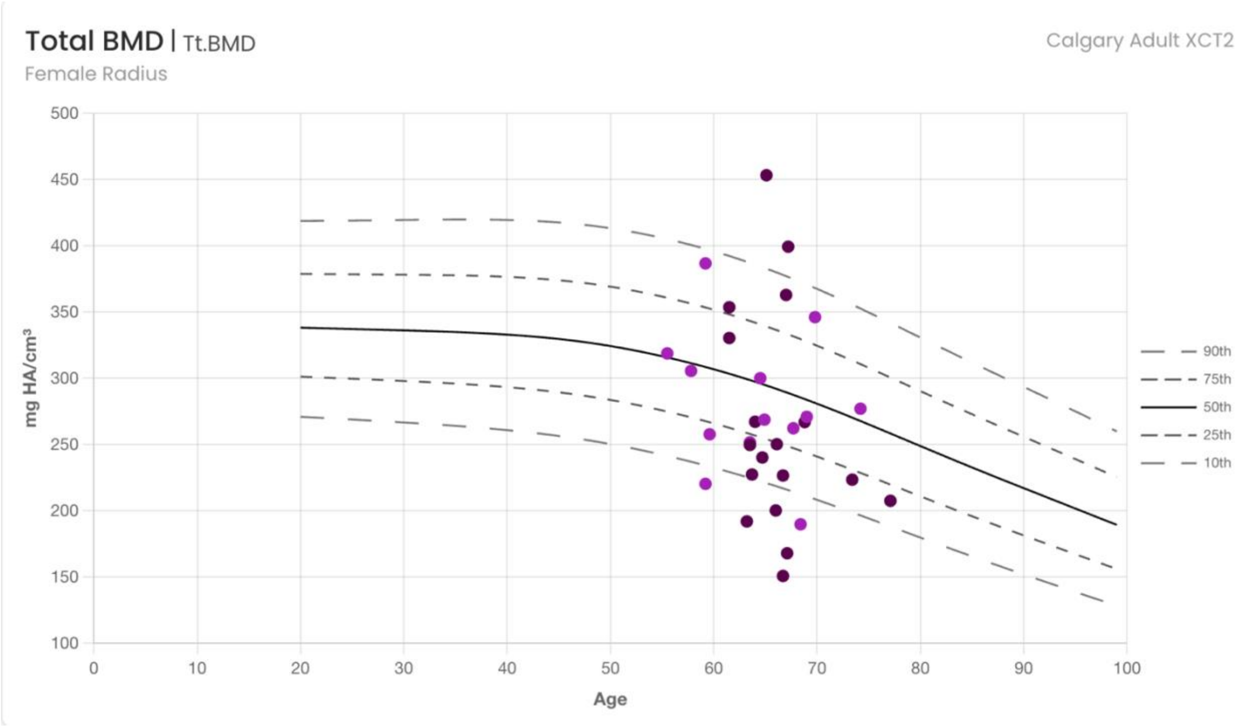

Supplementary Figure 1: Female radius total BMD. The inflammatory group is in light purple and the stricturing group is in dark purple.

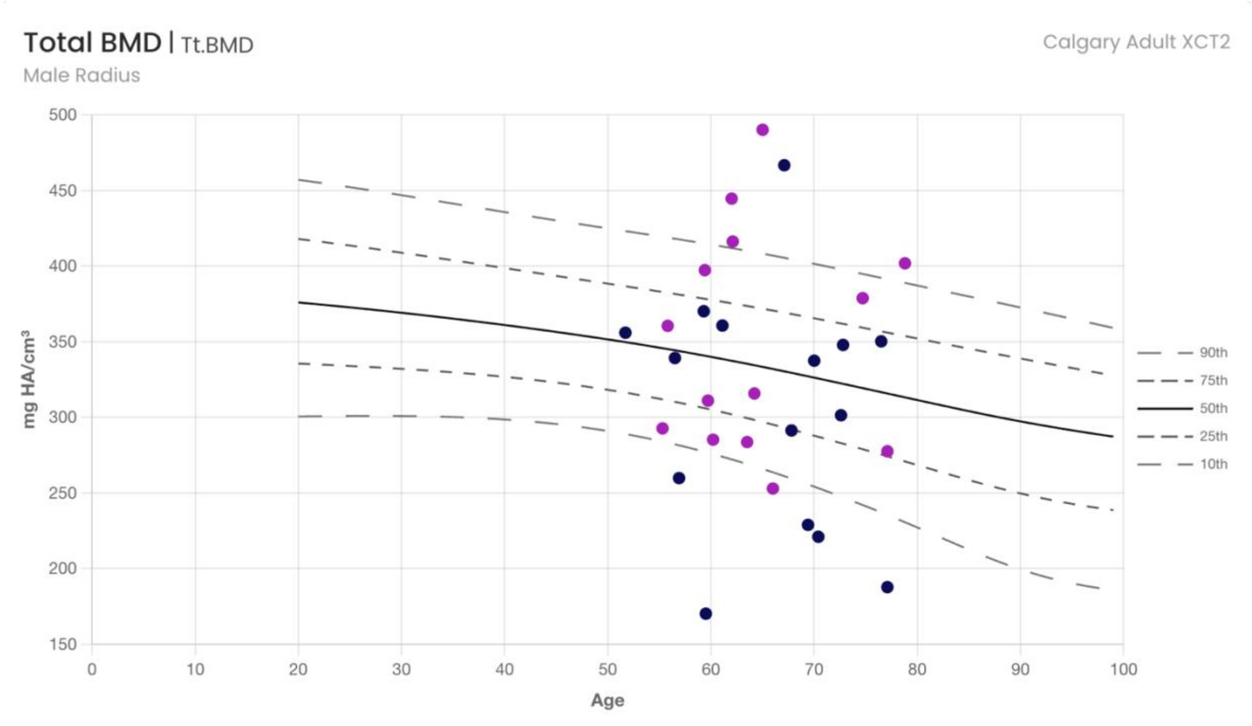

Supplementary Figure 2: Male radius total BMD. The inflammatory group is in light purple and the stricturing group is in blue.

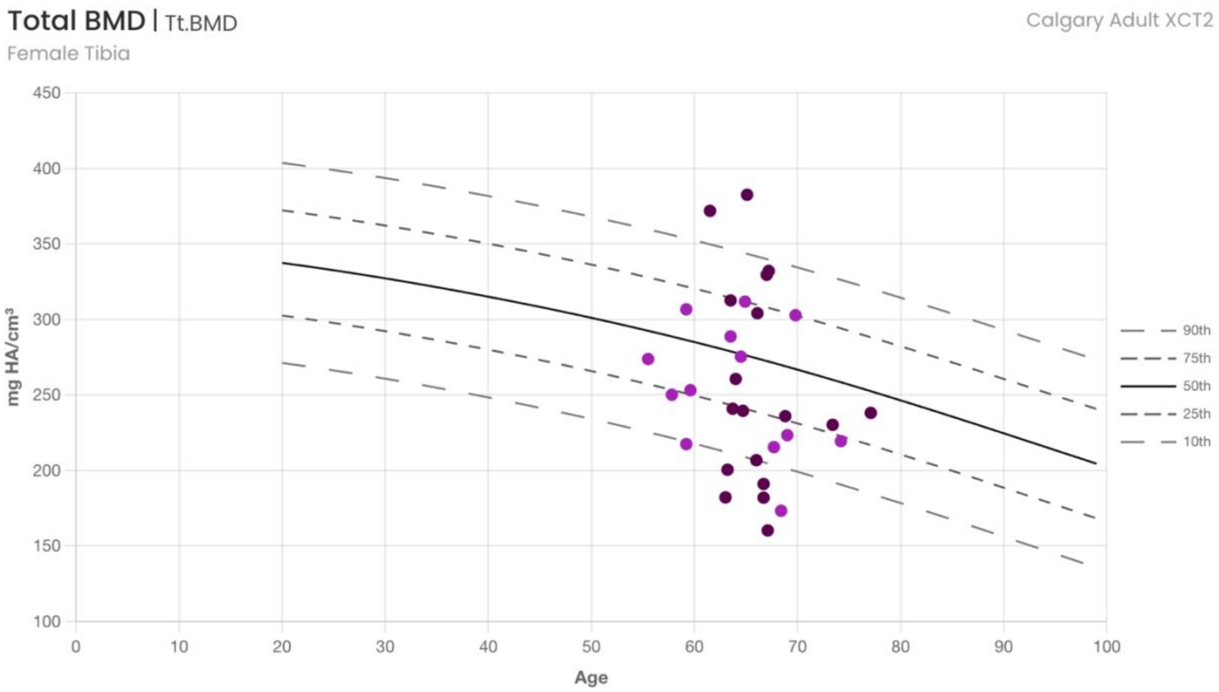

Supplementary Figure 3: Female tibia total BMD. The inflammatory group is in light purple and the stricturing group is in dark purple.

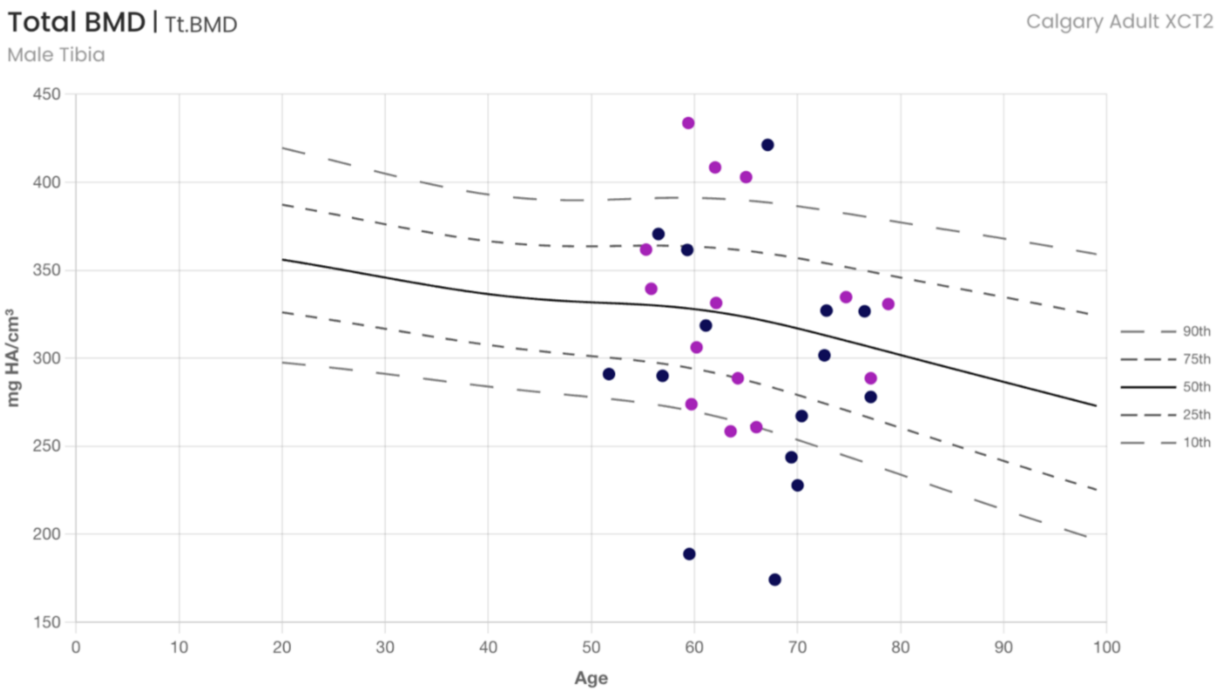

Supplementary Figure 4: Male tibia total BMD. The inflammatory group is in light purple and the stricturing group is in blue.

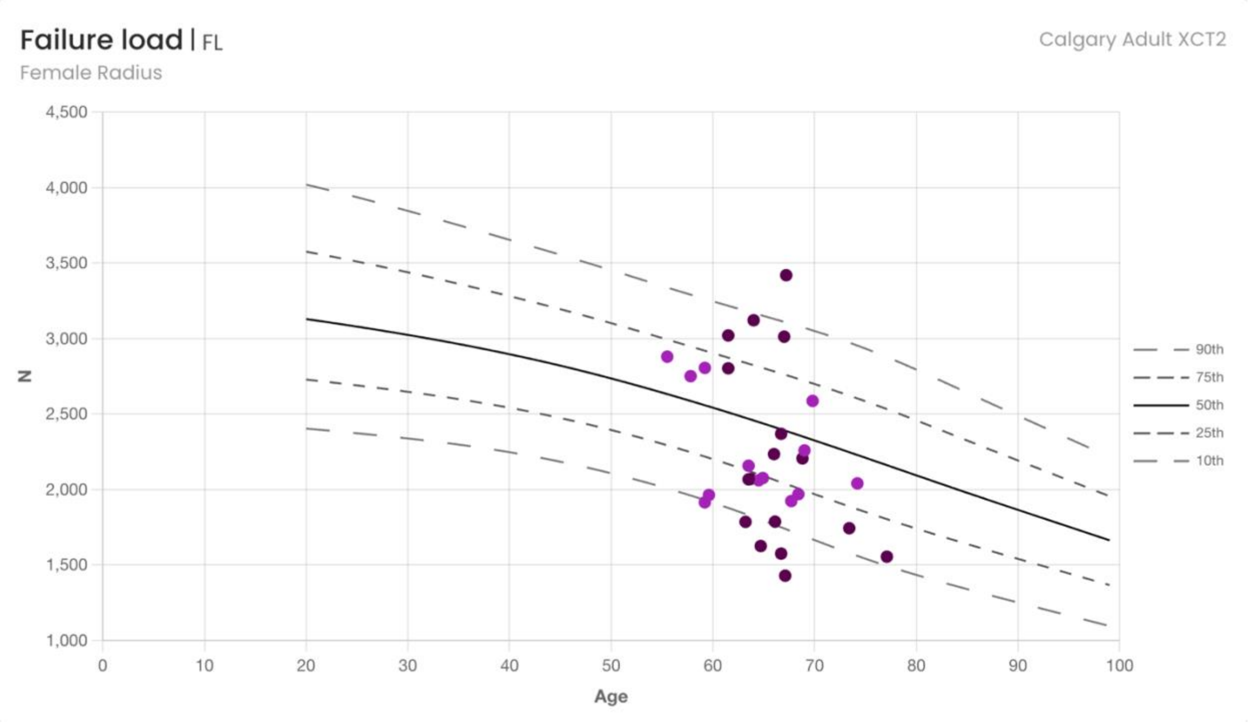

Supplementary Figure 5: Female radius failure load. The inflammatory group is in light purple and the stricturing group is in dark purple.

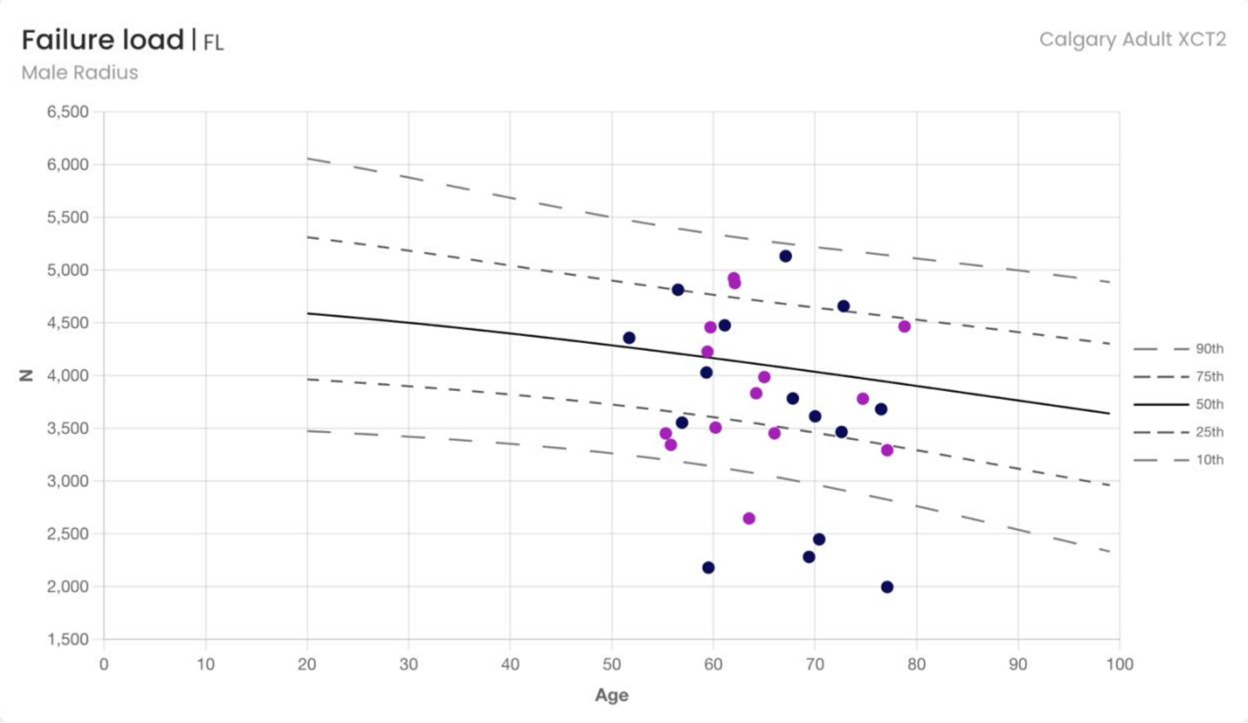

Supplementary Figure 6: Male radius failure load. The inflammatory group is in light purple and the stricturing group is in blue.

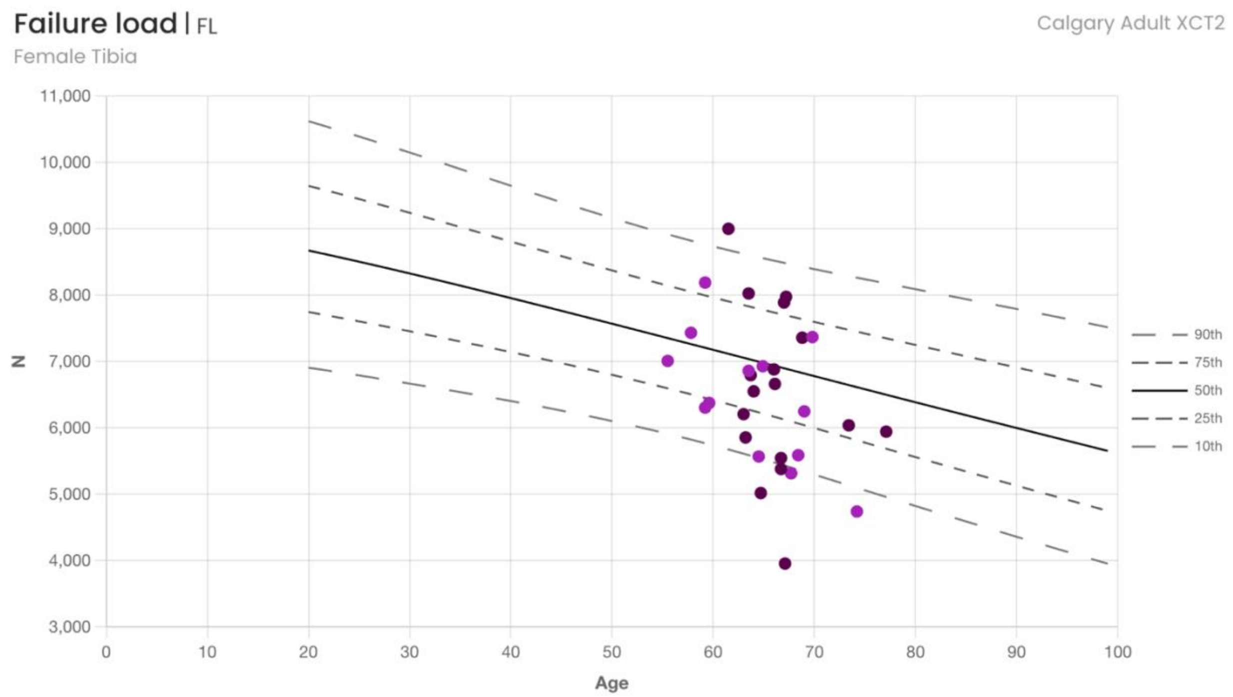

Supplementary Figure 7: Female tibia failure load. The inflammatory group is in light purple and the stricturing group is in dark purple.

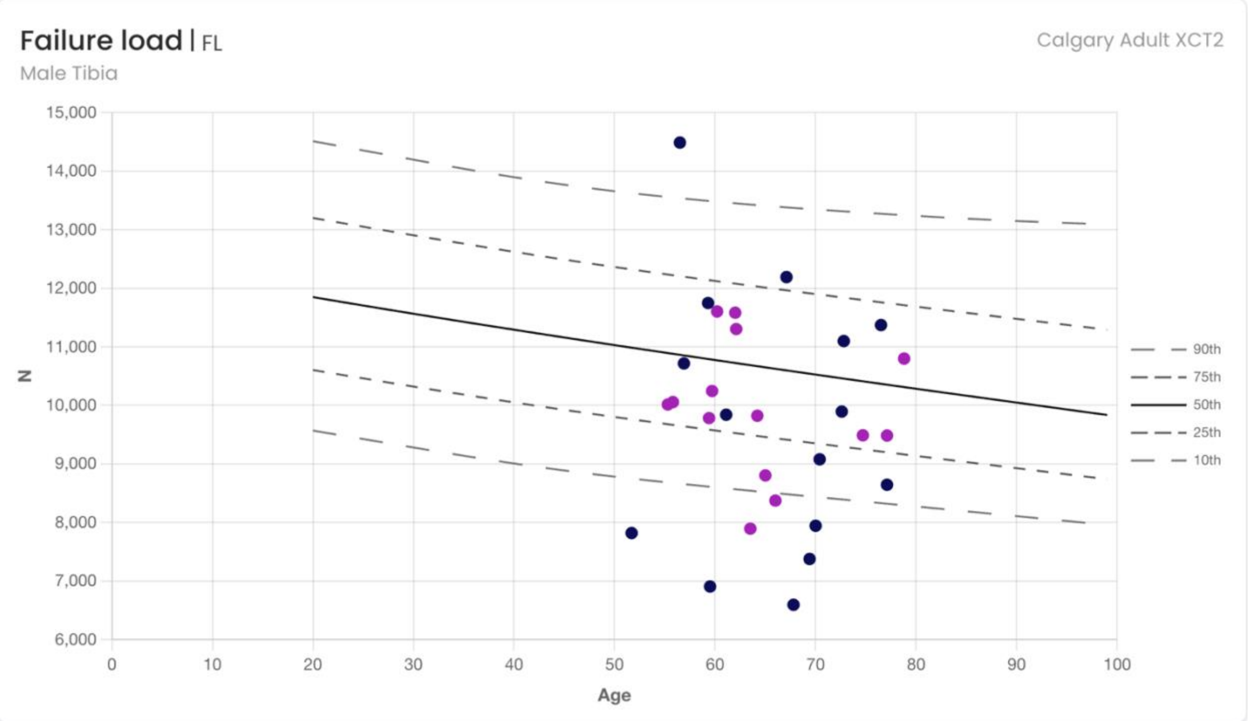

Supplementary Figure 8: Male tibia failure load. The inflammatory group is in light purple and the stricturing group is in blue.
